# Epidemiological Characteristics of Hypertension in the Elderly in Beijing, China

**DOI:** 10.1371/journal.pone.0135480

**Published:** 2015-08-21

**Authors:** Lina Ma, Xiaoling Zhao, Zhe Tang, Yun Li, Fei Sun, Lijun Diao, Gaizhen Ge, Ming Feng, Jieyu Wang

**Affiliations:** 1 Department of Geriatrics, Xuan Wu Hospital, Capital Medical University, Beijing, China; 2 Department of Epidemiology and Social Medicine, Xuan Wu Hospital, Capital Medical University, Key Laboratory for Neurodegenerative Disease of Ministry of Education, Center of Alzheimer's Disease, Beijing Institute for Brain Disorders, Beijing, China; 3 Department of Emergency, Three Gorges University People's Hospital, The First People's Hospital Of Yichang, Yichang, China; National Cardiovascular Center Hospital, JAPAN

## Abstract

**Background/Objectives:**

The prevalence rate of hypertension increases significantly with the aging society, and hypertension is obviously becoming a major health care concern in China. The aim of the study was to explore the epidemiological characteristics of hypertension in the elderly and to provide a basis for the prevention of hypertension.

**Design:**

3-cross sectional studies in 2000, 2004, and 2007, respectively.

**Setting:**

Beijing, China.

**Participants:**

A group of 2,832, 1,828, and 2,277 elderly residents aged ≥60 years were included this study in 2000, 2004, and 2007, respectively.

**Intervention:**

None.

**Measurements:**

Statistical sampling techniques included cluster, stratification, and random selection. Trained staff used a comprehensive geriatric assessment questionnaire and a standard survey instrument to complete the assessments. During the person-to-person interviews, the participants’ demographic characteristics, living conditions, and health status were collected, and their blood pressure was measured.

**Results:**

The prevalence rates (69.2%, 61.9%, and 56.0%) of hypertension and the control rates (22.6%, 16.7%, and 21.5%) lowered annually, while the awareness rates (43.7%, 55.8%, and 57.6%) of the treatment elevated annually in 2000, 2004, and 2007, respectively. There was no increase in the control rates for males (26.2%, 16.7%, and 20.8%), younger participants (28.0%, 18.4%, and 21.0%), and rural residents (19.5%, 9.6%, and 13.4%) in 2000, 2004, and 2007, respectively.

**Conclusions:**

Our study findings indicated that the prevalence of hypertension is high in rural elderly participants, while the rates of awareness, treatment, and control were low. This suggests that effective public measures need to be developed to improve the prevention and control of hypertension.

## Introduction

Hypertension is the most important risk factor for cardiovascular disease [1]. The prevalence rate of hypertension increases significantly with the aging society, and the danger of cardiovascular and cerebrovascular events in elderly patients with hypertension increases more significantly than that in younger people; thus, the prevention and treatment of hypertension in the elderly are very important. There are >200 million hypertensive patients in China [2]. According to the third National Health Services Survey in 2003, the direct economic burden to China caused by hypertension was 20,150 million yuan, and the economic burden of coronary disease and stroke caused by hypertension is was up to 19,080 million yuan [3]. According to the National Nutrition and Health Survey in 2002, the awareness, treatment, and control rates were 26.8%, 21.3%, and 3.9%, respectively [4].

Hypertension is a multifactorial disease, and it is related to heredity, diet, environment pollution, and other factors. The incidence rate of hypertension is increasing annually, and it has become a serious threat to human health [5]. A British survey of 5,043 patients found that in elderly people aged ≥65 years, the prevalence rate of hypertension was 81%, treatment rate was 56%, and control rate was 19%; the control rates were 36% and 30% in males and females, respectively [6]. In 1991, a national sample survey in China showed that the prevalence rate of hypertension was 40.4% in those ≥60 years [7]. In 2002, the prevalence rate of hypertension in the Liaoning Province was 59% in those aged ≥65 years; in 2003, the prevalence rate of hypertension was 80% in those aged ≥60 years; and in 2005, the prevalence rate of hypertension was 60.2% in those aged ≥60 years [8–10]. In order to observe the prevalence, awareness, treatment, and control rates of hypertension in the elderly population in Beijing, China, we conducted 3-cross sectional surveys in 2000, 2004, and 2007, respectively.

## Methods

### Study sample

**Fig 1:**
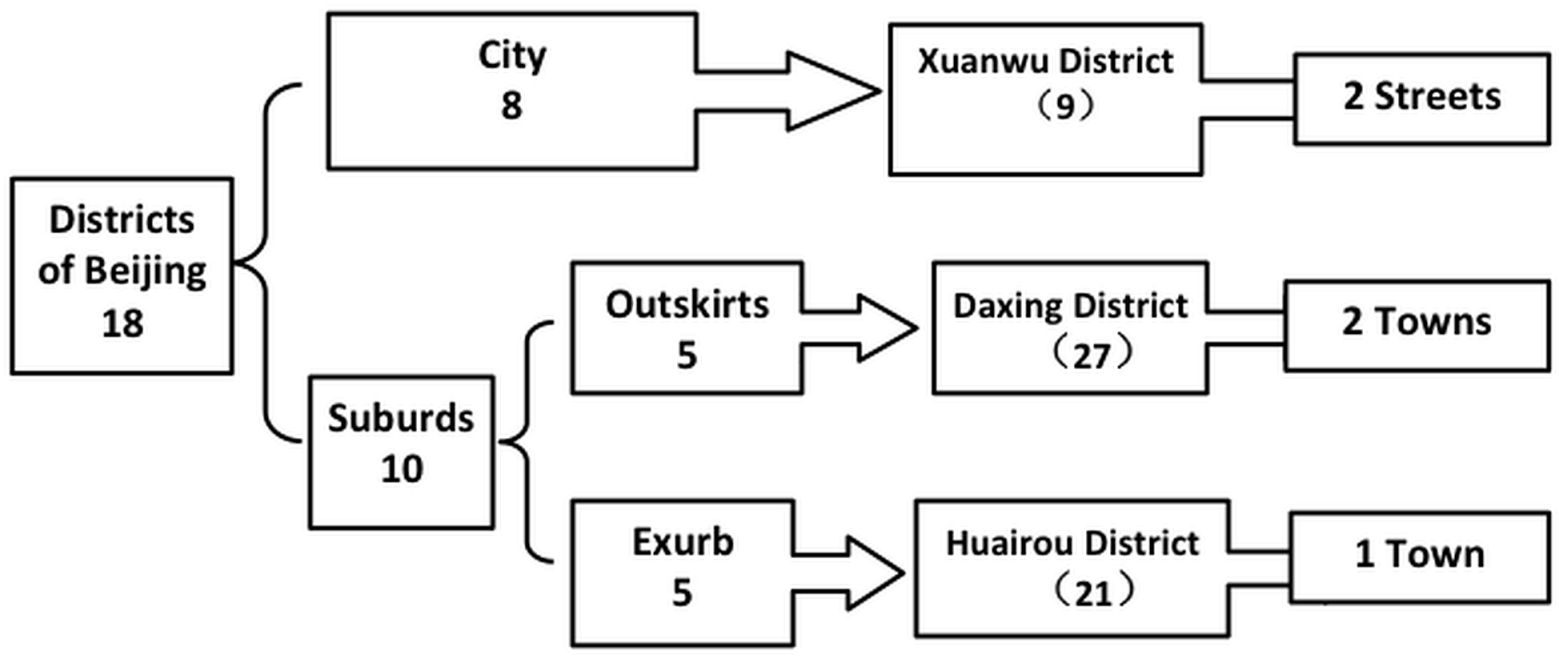
The sampling of research project. Specific approach is divided into three steps. The first step, according to the natural and living environment and economic level, 18 administrative districts were divided into two categories: City (8 districts) and suburds districts (10 districts, including 5 Outskirts plain districts and 5 exurds mountain districts). Then from the above two areas, 3 districts (Xuanwu District, Daxing District, Huairou District) were selected in three districts as representative of middle economic level. The above three districts were composed of 9 streets, 27 towns and 21 towns seperately. The second step, according to the population aging degree and culture degree, a random sample of 2 streets in Xuanwu District, 2 towns in Daxing District, 1 village in Huairou District was selected. Several neighborhood committees and villages were randomly selected from the above districts. The third step, in the selected neighborhood committees and villages, all aged 60 years and older were registered and arranged to several age groups according to age, 60~64 years group, 65~69 years group, 70~74 years group, 75~79 years old group and 80 ~ group. The number of each age group in identified each town, neighborhood committee and village was determined according to the distribution proportion of the existing population of Beijing.

Data for these analyses were obtained from the Beijing Longitudinal Study of Aging [11,12]. The project baseline was based on sample data from the fourth census of Beijing, China. Sampling was obtained from a city district (the Xuanwu District), an outskirt (the Daxing District), and an exurb area (the Huairou District) using well-established statistical sampling techniques, including cluster, stratification, and random selection. The sampling scheme is shown in Fig 1. 2972, 2104 and 2567 individuals were surveyed in the year 2000, 2004 and 2007, and the participation rate was 95.3%, 86.9% and 88.7%, respectively. The reasons of rejection of participation were not cooperate with the investigation, go out not at home and so on. This study included 2,832, 1,828, and 2,277 people aged ≥60 years in communities of Beijing, China in 2000, 2004, and 2007, respectively. The study was approved by the Xuan Wu Hospital’s Committee on Ethics of Human Experiments. All study participants provided written informed consent prior to enrollment. The participant consent was recorded in a file and the ethics committee approved this consent procedure.

### Data collection

Trained staff completed the questionnaires by using standard survey instruments. During the person-to-person interviews, data were collected on the participants’ demographic characteristics, living conditions, and health status, and their blood pressure was measured.

### Measurement of blood pressure

The blood pressure of each participant was measured in a quiet environment by trained doctors. Investigators were trained by the professional staff. The blood pressure was obtained from the nondominant arm with a mercury sphygmomanometer, which was calibrated regularly every three months. Two sitting blood pressure readings were taken on the right arm after 5 min of rest. Systolic and diastolic blood pressures were Korotkoff I and V, respectively, and an average was taken of the two values. The same machines were used in different sites and different years. The device was calibrated at the beginning of the recording by inducing stepwise changes in pressure from 0 to 200 mm Hg through the device pump, which was connected to a mercury column [13]. Hypertension was defined as systolic blood pressure (SBP) ≥140 mm Hg, diastolic blood pressure (DBP) ≥90 mm Hg, current treatment with antihypertensive medication, or a self-reported diagnosis of hypertension. The individuals were screened for a definitive diagnosis of hypertension depending on whether they were receiving antihypertensive drugs.

### Evaluation of demographic characteristics of hypertension

Participants who reported that a doctor or another health worker ever told them they had hypertension were considered aware of their disease, and the awareness rate of hypertensive was defined as the percentage of patients who knew they had hypertension before the investigation. The treatment rate was defined as the percentage of those among the hypertensive patients who were taking antihypertensive drugs in the last two weeks. Participants who were using antihypertensive medication and who had both SBP and DBP pressure lower than 140/90 mm Hg were classified as having controlled hypertension,and the control rate was defined as the percentage of those among the hypertensive patients whose who had controlled hypertension [4,14].

### Data quality control

Investigators received unified training. Two reviewers independently evaluated and crosschecked the questionnaire. The file management method was used to ensure the authenticity and homogeneity of data. Quality control points in the process of implementation was set up, and manual check was conducted to ensure the authenticity and integrity of data.

### Statistical methods

All the statistical analyses, including the χ^2^ tests, were performed using SPSS, version 11.5 (SPSS, Inc., Chicago, IL, USA). A P value <0.05 was considered statistically significant.

## Results

### The epidemiological characteristics of hypertension in the elderly in Beijing

There was a decreasing tendency in the prevalence rate of hypertension in the elderly in Beijing, and the prevalence rates were 69.2%, 61.9%, and 56% in 2000, 2004, and 2007, respectively. The awareness and treatment rates of hypertension increased, while the control rate of hypertension did not improve. In 2007, the prevalence rate of hypertension was 56%, and the awareness, treatment, and control rates were 57.6%, 56.2%, and 21.5%, respectively, in the elderly population in Beijing (Table 1).

**Table 1:** The epidemiological trend of hypertension in the elderly in Beijing 2000–2007.

Year	Number	Prevalence rate [Number (%)]	Awareness rate [Number (%)]	Treatment rate [Number (%)]	Control rate [Number (%)]
**2000**	2832	1960(69.2)	856(43.7)	694(35.4)	442(22.6)
**2004**	1828	1131(61.9)	631(55.8)	540(47.7)	189(16.7)
**2007**	2277	1275(56.0)	734(57.6)	716(56.2)	274(21.5)
*x* ^2^		95.7	75.3	140.4	15.4
*P*		<0.001	<0.001	<0.001	<0.001

### The epidemiological characteristics of hypertension in the elderly according to sex, age, and living condition

The prevalence rate of hypertension in men and women were decreased, and the awareness and treatment rates were improved; however, the control rate of hypertension did not increase (Table 2).

**Table 2:** The epidemiological trend of hypertension in the elderly in different gender, age and area.

Gender	Year	Number	Prevalence rate [Number (%)]	Awareness rate [Number (%)]	Treatment rate [Number (%)]	Control rate [Number (%)]
**Male**	2000	1380	917(66.4)	360(39.3)	295(32.2)	240(26.2)
	2004	882	527(59.8)	273(51.8)	232(44.0)	88(16.7)
	2007	1052	523(49.7)	271(51.8)	264(50.5)	109(20.8)
	*x* ^2^		69.4	31.3	50.8	18.0
	*P*		<0.001	<0.001	<0.001	<0.001
**Female**	2000	1452	1043(71.8)	496(47.6)	399(38.3)	202(19.4)
	2004	946	604(63.8)	358(59.3)	308(51.0)	101(16.7)
	2007	1225	752(61.4)	463(61.6)	452(60.1)	165(21.9)
	*x* ^2^		35.6	41.6	85.9	5.9
	*P*		<0.001	<0.001	<0.001	0.051
**Younger**	2000	1758	1217(69.2)	566(46.5)	463(38.0)	341(28.0)
	2004	991	609(61.5)	360(59.1)	321(52.7)	112(18.4)
	2007	1331	696(52.3)	412(59.2)	391(56.2)	146(21.0)
	*x* ^2^		92.1	41.0	70.6	24.6
	*P*		<0.001	<0.001	<0.001	<0.001
**Older**	2000	1074	743(69.2)	290(39.1)	231(31.1)	101(13.6)
	2004	837	522(62.4)	271(51.9)	219(42.0)	77(14.8)
	2007	946	579(61.2)	322(55.6)	325(56.1)	128(22.1)
	*x* ^2^		16.4	40.9	83.8	19.3
	*P*		<0.001	<0.001	<0.001	<0.001
**Rural**	2000	1298	917(70.6)	318(34.7)	236(25.7)	179(19.5)
	2004	976	612(62.7)	305(49.8)	235(38.4)	59(9.6)
	2007	1389	755(54.4)	358(47.4)	333(44.1)	101(13.4)
	*x* ^2^		75.9	44.1	65.0	29.8
	*P*		<0.001	<0.001	<0.001	<0.001
**City**	2000	1534	1043(68.0)	538(51.6)	458(43.9)	263(25.2)
	2004	852	519(60.9)	326(62.8)	305(58.8)	130(25.0)
	2007	888	520(58.6)	376(72.3)	383(73.7)	173(33.3)
	*x* ^2^		25.2	64.9	127.9	14.4
	*P*		<0.001	<0.001	<0.001	<0.001

The patients were divided into two groups according to age: younger (aged <75 years) and older (>75 years). Our study found that the prevalence rate of hypertension was decreased in both groups, while the awareness and treatment rates were improved in both groups. The control rate in the elderly group was increased, while the control rate in the younger group did not improve (Table 2).

The prevalence rate of hypertension decreased in the rural and city groups, and the awareness and treatment rates were increased in both groups. The control rate was improved in the city group; however, it was not improved in the rural group (Table 2).

## Discussion

Our study showed there was a great deal of variability in some of the prevalences between years, although we used the same way to collect data, the same sampling techniques, the same trained investigators and the same questionnaires between years, and the standard survey instruments were used to avoid the variablity. This study showed that the prevalence rate of hypertension in the elderly decreased annually, which indicates that the prevention of hypertension was greatly improved. There is a great progress in hypertension health education thses years in China, and those people who were in the high normal blood pressure can take active measures to prevent the progress of prehypertensive into hypertension. The popularity of hypertension prevention knowledge in this community makes it easier to diagnose and treat the elderly with hypertension in Beijing’s urban and rural areas; thus, the awareness and treatment rates were significantly improved. In 1991, the ratio of the controlled to treated individuals was 1:4, and this remained mostly unaltered in 2002 at 1:4.2 [4]; in our study, the ratio remained at 1:4.4, 1:5.9, and 1:4.6 in 2000, 2004, and 2007, respectively. Compared with many other countries, the ratio of the controlled to treated individuals was almost the same (about 1:3); however, it was obviously much lower than that in the United States(about 2:3) [15–18]. The control rate over the 7 years in our study did not significantly increase or even decline, and the reason may lie in the unstandardized treatment of hypertension and the decreased drug compliance, which led to the unsatisfactory degree of blood pressure control.

Many factor such as aging in China, genetic, environment, behaviour and ecology of the medicare on aging and hypertension caused the above phenomenon. China will experience an enormous increase in the prevalence of cardiovascular-related morbidity and mortality that is attributable to blood pressure over the next few years [4]. Another study performed in the Henan Province of China reported that the levels of hypertension awareness, treatment, and control are low, which indicates that necessary actions such as prevention, detection, and treatment are necessary to prevent the situation from worsening [19]. Some studies had found polymorphism of the specific gene was associated with cardiovascular risk factors and may contribute to susceptibility to cardiovascular disease [20], while some other studies found there was no significant association identified between GNB3-C825T polymorphism and EH in Han Chinese population [21]. Chinese Government has initiated widespread reform [22], reinforce health promotion in older adults and improve health services in rural communities [23]. Shao S attempted to map the medical care ecology of Beijing urban population and provides timely baseline information for health care reform in China [24].

Our study indicated that the state of prevention and control of hypertension in the elderly in Beijing, China is not good, especially in the younger, rural hypertensive population. Individuals with hypertension have lower quality of life than normotensives in China, even after controlling for gender and age [25]. This study showed that the control rate did not improve in the younger group, there are several reasons, some younger patients are young is still at full time or part time work, and increased mental stress, which lead to poor blood pressure control; therefore, we should strengthen the health care education for this population, encourage them to change their life style, monitor their blood pressure, and support regular attendance at follow-ups. The control rate did not increase in the rural population, which may be due to the difference between the urban and rural living standards, which include the dietary habits, physical labor intensity, and mental pressure [26,27]. People in the rural areas may have a lower education level, lack of health care knowledge, and absence of self-health care consciousness, which can lead to a low control rate of blood pressure; therefore, we should pay attention to the prevention and control of hypertension in the rural population.

A limitation of our study was that the sample was restricted to community residents in Beijing, China. Therefore, our findings are not representative of the overall Chinese population. The numbers of participant is moderate, not very large, and the study was respective not continuous. Therefore, a large scale analysis comprising continuous study would be desirable.

## Conclusion

Hypertension is a lifelong disease, and long-term treatment is needed; therefore, we should strengthen the prevention and control of hypertension and health care knowledge in the Beijing community. It is important to establish health files in the community; make full use of the health education and health promotion interventions; encourage people to change their attitude on hypertension; and constantly improve the awareness, treatment, and control rates of hypertension in order to effectively control hypertension and to reduce the incidence and mortality of cardiovascular diseases, which will improve the overall health level of this population [28,29].

## Supporting Information

S1 File The data underlying our findings. The file shows all the data included 2,832, 1,828, and 2,277 people aged ≥60 years in communities of Beijing, China in 2000, 2004, and 2007, respectively.
(SAV)

## References

[1] World Organization. The world health report 2002-reducing risks, promoting healthy life. *Educ Health (Abingdon)*. 2003; 16: 230. 14741909 10.1080/1357628031000116808

[2] Huang Y, Qiu W, Liu C, Zhu D, Hua J, Cai X, et al Prevalence and risk factors associated with prehypertension in Shunde District, southern China. *BMJ Open*. 2014; 4: e006551. 10.1136/bmjopen-2014-006551 PMC4244395 25394820

[3] Kim JS. Stroke in Asia: a global disaster. *Int J Stroke*. 2014;9:856–857. 25231579 10.1111/ijs.12317

[4] Wu Y, Huxley R, Li L, Anna V, Xie G, Yao C, et al Prevalence, awareness, treatment, and control of hypertension in China: data from the China National Nutrition and Health Survey 2002. *Circulation*. 2008;118:2679–2686 19106390 10.1161/CIRCULATIONAHA.108.788166

[5] Mancia G, Giannattasio C. Diagnostic and therapeutic problems of isolated systolic hypertension. *J Hypertens*.2015; 33:33–43. 25426565 10.1097/HJH.0000000000000424

[6] Paola P, Neil RP. Hypertension management and control among English adults aged 65 years and older in 2000 and 2001. *J Hypertens*. 2004; 22: 1093–1098. 15167442 10.1097/00004872-200406000-00008

[7] Liang Y, Liu R, Du S, Qiu C. Trends in incidence of hypertension in Chinese adults, 1991–2009: the China Health and Nutrition Survey. *Int J Cardiol*. 2014;175:96–101. 24833472 10.1016/j.ijcard.2014.04.258 PMC4105139

[8] Li X, Li S, Jin X, et al Prevalence of hypertension in Liaoning residents. *Chin Chronic Prevent Control*. 2006; 14: 94–97.

[9] Chen X, Wei W, Zou S, Wu X, Zhou B, Fu L, et al Trends in the prevalence of hypertension in island and coastal areas of china: a systematic review with meta-analysis. *Am J Hypertens*. 2014;27:1503–1510. 24610901 10.1093/ajh/hpu026

[10] Huang Y, Qiu W, Liu C, Zhu D, Hua J, Cai X, et al Prevalence and risk factors associated with prehypertension in Shunde District, southern China. *BMJ Open*. 2014; 4:e006551. 10.1136/bmjopen-2014-006551 PMC4244395 25394820

[11] Wang C, Song X, Mitnitski A, Fang X, Tang Z, Yu P, et al Effect of health protective factors on health deficit accumulation and mortality risk in older adults in the Beijing Longitudinal Study of Aging. *J Am Geriatr Soc*. 2014;62:821–828. 24749784 10.1111/jgs.12792

[12] Zimmer Z, Fang X, Tang Z. Fifteen-year disability trends among older persons in the Beijing municipality of China. *J Aging Health*. 2014;26: 207–230. 24336232 10.1177/0898264313513609

[13] Parati G, Ulian L, Santucciu C, Omboni S, Mancia G. Difference between clinic and daytime blood pressure is not a measure of the white coat effect. *Hypertension*. 1998; 31:1185–1189. 9576133 10.1161/01.hyp.31.5.1185

[14] Supiyev A, Kossumov A, Utepova L, Nurgozhin T, Zhumadilov Z, Bobak M. Prevalence, awareness, treatment and control of arterial hypertension in Astana, Kazakhstan. A cross-sectional study. *Public Health*. 2015 3 25 pii: S0033-3506(15)00069-4. doi: 10.1016/j.puhe.2015.02.020. [Epub ahead of print] 10.1016/j.puhe.2015.02.020 25818013

[15] Kaplan NM, Opie LH. Controversies in hypertension. *Lancet*. 2006; 367: 168–176. 16413880 10.1016/S0140-6736(06)67965-8

[16] Weinehall L, Ohgren B, Persson M, Stegmayr B, Boman K, Hallmans G, et al High remaining risk in poorly treated hypertension: the “rule of halves” still exists. *J Hypertens*. 2002; 20: 2081–2088. 12359988 10.1097/00004872-200210000-00029

[17] Joshi SR, Shah SN. Control of blood pressure in India: rule of halves still very much valid. *J Assoc Physicians India*. 2003; 51: 151–152. 12725256

[18] Ong KL, Cheung BMY, Man YB, Lau CP, Lam KS. Prevalence, awareness, treatment, and control of hypertension among United States adults 1999–2004. *Hypertension*. 2007; 49: 69–75. 17159087 10.1161/01.HYP.0000252676.46043.18

[19] Fan L, Feng SX, Han B, Wang CC, Gao L, Feng HF, et al Prevalence, awareness, treatment and control of hypertension in Henan Province, China. *Aust J Rural Health*. 2014;22:264–269. 25303419 10.1111/ajr.12116

[20] Yan YX, Dong J, Wu LJ, Shao S, Zhang J, Zhang L, et al Associations between polymorphisms in the glucocorticoid-receptor gene and cardiovascular risk factors in a Chinese population. *J Epidemiol*. 2013;23:389–395. 23892712 10.2188/jea.JE20130035 PMC3775534

[21] Lu J, Guo Q, Zhang L, Wang W. Association between the G-protein β3 subunit C825T polymorphism with essential hypertension: a meta-analysis in Han Chinese population. *Mol Biol Rep*. 2012;39:8937–8944. 22714918 10.1007/s11033-012-1762-1

[22] Ling RE, Liu F, Lu XQ, Wang W. Emerging issues in public health: a perspective on China's healthcare system. *Public Health*. 2011;125:9–14. 21168175 10.1016/j.puhe.2010.10.009

[23] Peng X, Song S, Sullivan S, Qiu J, Wang W. Ageing, the urban-rural gap and disability trends: 19 years of experience in China—1987 to 2006. *PLoS One*. 2010;5:e12129. 20730089 10.1371/journal.pone.0012129 PMC2921329

[24] Shao S, Zhao F, Wang J, Feng L, Lu X, Du J, et al The ecology of medical care in Beijing. *PLoS One*. 2013;8:e82446. 24340029 10.1371/journal.pone.0082446 PMC3855438

[25] Liang XY, Nie SF, Qu KY, Peng XX, Wei S, Zhu GB, et al Evaluation of health-related quality of life among hypertensive patients in a rural area, PR China. *J Hum Hypertens*. 2006;20:227–229. 16341052 10.1038/sj.jhh.1001967

[26] Yang L, Xu X, Yan J, Yu W, Tang X, Wu H, et al Analysis on associated factors of uncontrolled hypertension among elderly hypertensive patients in Southern China: a community-based, cross-sectional survey. *BMC Public Health*. 2014;14:903. 25178313 10.1186/1471-2458-14-903 PMC4247067

[27] Su D, Du H, Zhang X, Qian Y, Chen L, Chen Y, et al Season and outdoor temperature in relation to detection and control of hypertension in a large rural Chinese population. *Int J Epidemiol*. 2014; 43:1835–1845. 25135908 10.1093/ije/dyu158 PMC4276060

[28] Cheng HM, Pearson A, Sung SH, Yu WC, Chen CH, Karnon J. Cost-effectiveness of noninvasive central blood pressure monitoring in the diagnosis of hypertension. *Am J Hypertens*. 2015;28: 604–614. 25430695 10.1093/ajh/hpu214

[29] Mancia G, Giannattasio C. Diagnostic and therapeutic problems of isolated systolic hypertension. *J Hypertens*. 2015;33:33–43. 25426565 10.1097/HJH.0000000000000424

